# Association between DHA and depression: results from the NHANES 2011–2014 and a bidirectional Mendelian randomization analysis

**DOI:** 10.1186/s40001-025-02918-4

**Published:** 2025-07-22

**Authors:** Yu-hang Chen, Yu Zhang, Wang Zhang

**Affiliations:** Chongqing Mental Health Center, No.102, Jinzi Mountain, Jiangbei District, Chongqing, 401147 China

**Keywords:** DHA, Depression, NHANES, Mendelian randomization, Association

## Abstract

**Background:**

A great deal of research demonstrates that the pathophysiology and etiology of depression have been associated with dietary deficiencies in omega-3 polyunsaturated fatty acids (n-3 PUFAs). However, little is known about this association’s common genetics and causal relationships. Therefore, we used observational studies combined with bidirectional Mendelian randomization (MR) to investigate a potential association between depression and docosahexaenoic acid (DHA).

**Methods:**

Using data from the National Health and Nutrition Examination Survey (NHANES) in the United States from 2011 to 2014, we first conducted a cross-sectional study and analyzed the association between DHA and depression using a statistical method to adjust for confounders in logistic regression. We subsequently utilized genome-wide association study (GWAS) data in the UK to determine the causal relationship between DHA and depression by a genetic approach to assess causality for MR analysis. We used inverse variance weighting (IVW) methods to obtain the majority of the bidirectional causal estimates of MR. We additionally performed sensitivity analyses to analyze the horizontal pleiotropy and heterogeneity of MR results.

**Results:**

This NHANES analysis has shown that DHA is associated with depression in American adults (OR = 0.996, 95% CI 0.993–0.999, *P* = 0.014). Bidirectional MR analysis demonstrated a significant causal relationship between DHA and depression in the European population (OR = 0.9, 95% CI 0.84–0.97, *P* = 0.006).

**Conclusions:**

This research provides new insights into the association between DHA and depression. This discovery needs to be validated in further prospective studies that require large sample sizes and sufficient follow-up time.

**Supplementary Information:**

The online version contains supplementary material available at 10.1186/s40001-025-02918-4.

## Introduction

Depression is a mood disorder with a course lasting more than 2 weeks. Globally, the disease impacts over 264 million people [1]. The characteristics of depression include sadness, feelings of worthlessness or guilt, changes in appetite, sleep problems, and, most seriously, suicidal thoughts [2]. Antidepressant medications are the major treatment for depression. While there is some clinical efficacy to this treatment [3, 4], meta-analyses have revealed limited rates of remission [5], and only among patients with major depressive disorder have there been clinically significant distinctions between antidepressants and placebo. Withdrawal symptoms due to discontinuation have been reported for almost all antidepressants, with an incidence as high as 42.9% [6]. Nutritional psychiatry is gaining acceptance as dietary supplements are less likely to cause adverse effects than medications and can more easily provide nutritional support through food intake. 

Unsaturated fatty acids (UFAs) are one of the indispensable nutrients in the human diet, and their role is mainly reflected in the promotion of neurodevelopment and the maintenance of brain function, but also closely related to behavioral and mental health [7, 8]. UFAs are categorized as monounsaturated fatty acids (MUFAs) and polyunsaturated fatty acids (PUFAs) based on the number and position of the double-bond carbon. Among the PUFAs are n-3 PUFAs, which include α-linolenic acid (ALA), eicosapentaenoic acid (EPA), and DHA [9]. A sufficient DHA or n-3 PUFA diet is necessary as one ages to preserve proper brain function [10]. From the 8 clinical studies showing the benefits of PUFAs in depression, we observed DHA of 0.4–0.8 g, with intakes ranging from 1 to 9 times per day over a dosing period of 3 weeks to 4 months. One of these studies, in which the subjects were pregnant women at various stages of pregnancy, also showed a beneficial effect of PUFAs supplementation on depression in pregnancy [11]. This indicates that most of the studies agreed that dietary supplements containing DHA help treat depression. However, according to some meta-analyses and randomized controlled trials (RCTs), DHA supplements have not shown efficacy in improving symptoms of depression compared to EPA [12]. The recent meta-analysis by Chang et al. [13] showed that supplementing DHA is more effective than EPA in improving depressive symptoms in elderly dementia patients. Mischoulon et al. [14] demonstrated that for patients with depression, lower doses of DHA (i.e., 1 g/d) may be more effective than higher doses of DHA (2 or 4 g/d). This phenomenon is difficult to explain.

Therefore, understanding the relationship between DHA and depression can help overcome the current challenges faced by the public health field and aid in the prevention and intervention procedures of depression in clinical diagnosis and treatment. The design and exploration of RCTs are the gold standard for demonstrating this relationship. RCTs aim to explore the causal effects of pre-existing diseases; However, the small sample size, limited external validity, short intervention duration, and ethical issues limit the implementation of RCTs. Observational studies require relatively less workload compared to RCTs; However, due to residual confounding bias, reverse causality, or undetected bias, the results provide weaker causal inference. At this point, we can partially address the limitations of confounding and reverse causality using genetic data, and provide more convincing evidence to explain, which is known as the potential causal effect of MR. This method estimates the causal relationship between the exposure and the outcome [15]. Compared to observational studies, genetic variants follow the Mendelian law of inheritance, which states that alleles are randomly assigned during gamete formation and remain unchanged after birth, regardless of known or unknown factors like individual behavior, socioeconomic status, and environmental exposures. This makes MR more chronologically rational in causal inference [16]. The evidence of MR studies lies between observational studies and RCTs, but is limited by potential weak instrumental variables (IVs) and genetic pleiotropy [15]. Therefore, these two research techniques in this study can complement each other and make up for the shortcomings of RCTs.

Based on the results of existing studies, the role of DHA in ameliorating depression is not clear, and there is a lack of correlation studies between a single DHA and depression. Therefore, to fill this gap, this study will combine the NHANES observational study with the bidirectional MR study to explore the causal relationship between DHA levels and depression.

## Materials and methods

### Study population in NHANES

**Fig. 1:**
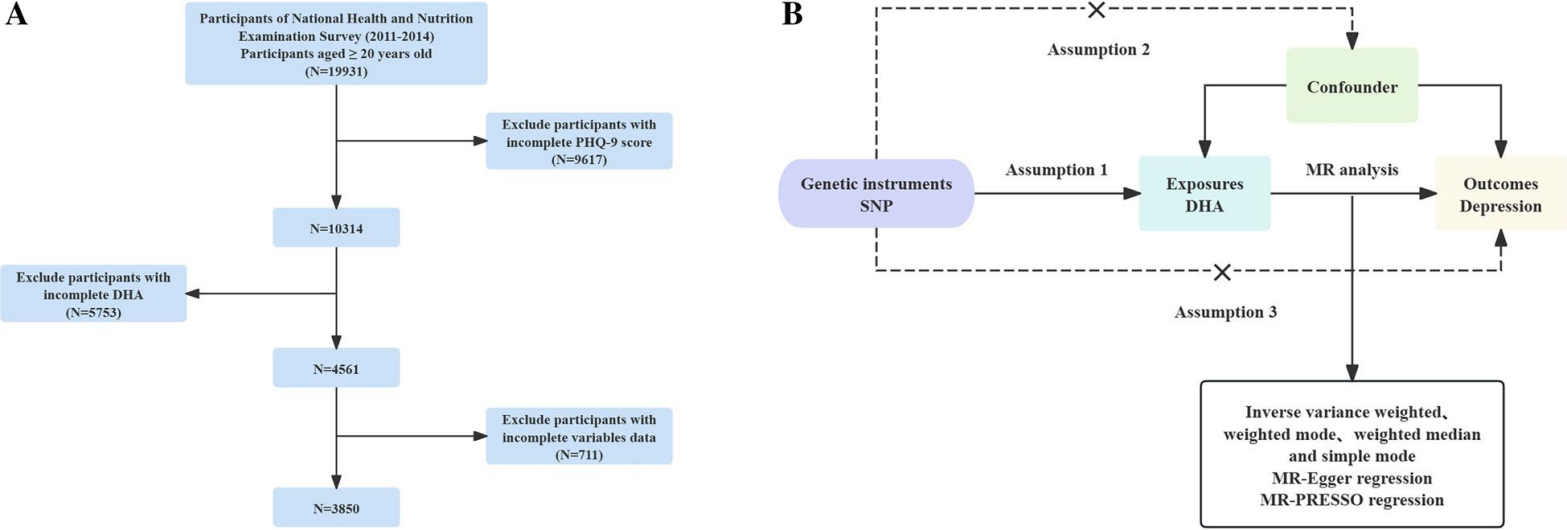
Overall study design based on observational analysis and MR. **A** Flow diagram of eligible participant selection in the NHANES. **B** Schematic diagram of the MR principle and procedures. National Health and Nutrition Examination Survey, NHANES; PHQ-9, Patient Health Questionnaire-9; MR, Mendelian randomization; SNPs, single-nucleotide polymorphisms

The Centers for Disease Control (CDC) and the National Center for Health Statistics (NCHS) conduct the nationally representative, cross-sectional NHANES survey to investigate the nutritional status and general health of the noninstitutionalized U.S. population [17]. The purpose of this survey is to evaluate the health and nutritional status of adults and children. It involves multiple data modules, including questionnaires, examinations in laboratories, physical examinations, and household surveys. Data have been released every 2 years since 1999. The National Center for Health Statistics Institutional Review Board has authorized NHANES, and each participant has provided written informed consent. Figure 1A shows the participant screening process.

According to NHANES data (https://www.cdc.gov/nchs/nhanes/index.htm), 19,931 participants were included in the two cycles from 2011 to 2014, ultimately limiting our analysis to 3,850 adults aged 20 years or older. Of these, we excluded participants with incomplete nine-item Patient Health Questionnaire (PHQ-9) depression scale (*n* = 9,617) and missing DHA data (*n* = 5,753).

### Definition of depression in NHANES

The PHQ-9 scale was used to assess the participants’ depressive status and measure the frequency of depression symptoms during the previous 2 weeks using nine items [18]. A total score ranging from 0 to 27 was obtained by assigning a four-point rating to each symptom item, ranging from 0 (“not at all”) to 3 (“almost every day”). According to prior research findings, depression was defined in this study as a PHQ-9 score of ≥10, while non-depression was defined as a score of <10 [19]. Although the PHQ-9 scale can classify the severity based on scores, it is currently not recommended to use n-3 PUFAs monotherapy to treat major depressive disorder (MDD), although supplementing them may be useful for certain specific populations [20]. Therefore, the correlation between DHA and MDD was not studied separately in this study.

### Assessment of DHA in NHANES

In this study, we utilized a subsample of the NHANES from 2011 to 2014, as that cycle covers information related to DHA. At the Division of Laboratory Sciences, U.S. CDC [21], over 30 subtypes of fatty acids (FAs), comprising 6 MUFAs, 13 PUFAs, and 17 saturated fatty acids (SFAs), were quantified and expressed in μmol/L in serum samples obtained after fasting using gas chromatography/mass spectrometry. To achieve accurate FAs recovery, total FAs were recovered from the matrix (100 μL serum or plasma) using hexane in conjunction with an internal standard solution. Following derivatization, the resultant extract was fed onto a capillary gas chromatograph column to create pentafluorobenzyl esters. The perfluoroalkyl ester–fatty acid derivatives were separated using electron capture negative ion mass spectrometry and capillary gas chromatography. Each FA class’s analyte recovery was assessed using the relevant internal standards. The NHANES handbook has more information regarding plasma FAs profile analysis.

### Ascertainment of covariates

Based on existing literature on PUFAs and depression, this study collected information on age, gender, race, education level, poverty-to-income ratio (PIR), alcohol and smoking habits, and body mass index (BMI) [22, 23].

### Genetic data and screening criteria for the MR analysis

Using the IEU Open GWAS website (https://gwas.mrcieu.ac.uk), pertinent GWAS data sets were obtained for this study. Genetic summary data for DHA were obtained from the Richardson TG study, a GWAS study sample that included a total of 115,006 European subjects and estimated the genetic association of 11,590,399 single-nucleotide polymorphisms (SNPs) with DHA, which is the largest data set in terms of sample size for this phenotype. To make the genetic characteristics of the two samples similar, databases with European populations were uniformly selected for the study, thus avoiding bias due to population stratification. Based on the above criteria, the latest and largest sample size GWAS was selected as the outcome sample. The genetic data details of DHA and depression are shown in Table S1.

Based on the genome-wide significance threshold (*P* < 5 × 10^−8^), SNPs were selected as IVs, indicating a substantial correlation between SNPs and DHA. To ensure the independence of SNPs, the coefficient of linkage disequilibrium (LD) was set in the R software (*r*^2^ < 0.01, window size = 10,000 kb). IV’s strength was assessed by calculating the *F* value of individual SNP to exclude possible weak IV bias between IV and exposure, with the following formula [24]:

$${R}^{2}=\frac{2\times \text{MAF}\times \left(1-\text{MAF}\right)\times {\beta }^{2}}{{\text{SE}}^{2}\times N}$$

$$F\text{-statistic}=\frac{{R}^{2}\times \left(N-k-1\right)}{{k}^{2}\left(1-{R}^{2}\right)}$$

A correlation between IV and exposure that is sufficiently high and minimizes the likelihood of weak IV bias is indicated by an F-statistic value larger than 10.

### Statistical analyses

In the baseline table, continuous variables are described using means and standard deviations, and categorical variables are expressed as percentages. We performed multivariable-adjusted logistic regression in the observational study with NHANES data to calculate odds ratios and 95% confidence intervals designed to assess the association between DHA levels and depression risk. DHA was examined for the interquartile group trend after being divided into quartiles. The lowest quartile (first quartile) was defined as the reference group in each model. The relationship between depression and the plasma DHA level quartiles has been analyzed using binary logistic regression analysis. Three multivariate regression models were constructed: crude, unadjusted; model 1, adjusted for age, gender, and race/ethnicity; model 2, adjusted for age, gender, race/ethnicity, education, RIP, BMI, smoking, and alcohol consumption.

Depression is the outcome variable of this study, and DHA is the exposure factor. The SNPs significantly associated with DHA is IVs. Three major hypotheses are required for MR research [25]: first, there is the relevance hypothesis (Hypothesis 1), which states that certain SNPs should be significantly correlated with the exposure factor; second, there is the independence hypothesis (Hypothesis 2), which states that SNPs must be independent of potential confounders between exposure and outcomes; and third, there is the exclusivity hypothesis (Hypothesis 3), which states that SNPs that are significantly correlated with exposure are not directly correlated with outcomes and can only be causally linked through exposure. Figure 1B shows the design of the MR research.

Five methods were utilized in this study to assess MR effect sizes: MR–Egger, simple mode, weighted mode, IVW, and weighted median. IVW, which assumes that all SNPs satisfy the three key assumptions of MR, involves analyzing the Wald ratios between exposure and outcome for each SNP, and then pooling the data to directly calculate the causal effect values. This method provides a more robust estimate of the causal effect. IVW analyses yielded the most reliable results when there was no horizontal multivalence for the IVs [26, 27]. Therefore, in this study, IVW was used as the main analytical method for MR. MR–PRESSO is a method specifically designed to detect horizontal pleiotropy [28]. It identifies SNPs that may have pleiotropy utilizing a global test and an outlier test. The global test assesses the presence of horizontal pleiotropy in all IVs, while the outlier test identifies specific outlier SNPs. When there is no pleiotropy (*P* > 0.05), it is considered that all SNPs included in the model are effective IVs. If the *p* ≤ 0.05, this indicates that the SNP is likely to be an outlier, may have pleiotropy, and should be removed from the IVs. Cochran’s Q is used to examine whether there is heterogeneity in the estimated SNPs proportion values in the model [29]. If there is heterogeneity between IVs, the result analysis will be based on the IVW of the random effects model. On the contrary, the IVW results of the fixed effects model are dominant [30].

To investigate whether depression has any causal effect on DHA, we also conducted reverse MR analysis using SNPs associated with depression as IVs. The IVs for reverse MR also had to fulfill the three major assumptions of IVs (correlation, independence, and exclusivity), that is, the selected IVs influenced exposure only through the outcome and not through other pathways. We used IVW as the primary method for studying reverse causality, and *P* > 0.05 indicated that the reverse MR results were not significant.

Statistical analyses for this study were conducted using R 4.3.2 software. In the NHANES study, data were described and statistically analyzed using the “Survey” package. *P* < 0.05 was considered statistically significant.

## Results

### Baseline characteristics

As shown in Table 1, a total of 3,850 U.S. adults participated in the NHANES 2011–2014 study. The weighted mean (standard error, SE) age of the study participants was 47.52 (0.47) years, and male and female participants were almost equally distributed genderwise. Among the participants, 1,712 (68.91%) were non-Hispanic white, 775 (10.80%) were non-Hispanic black, 454 (7.93%) were Mexican–American, 355 (5.55%) were Other Hispanic, and 554 (6.82%) were other race/ethnicity. Approximately 84.62% of respondents had completed at least high school/GED. Notably, participants with lower DHA levels were more likely to suffer from depression.

**Table 1:** Weighted baseline characteristics of participants in DHA quartiles

Characteristics	DHA (μmol/l)					*p* value
	Total	Q1 (25.7, 109)	Q2 (109, 145)	Q3 (145, 198)	Q4 (198, 918)	
	(*n* = 3850)	(*n* = 974)	(*n* = 963)	(*n* = 955)	(*n* = 958)	
Age (years)	47.52 (0.47)	41.20 (0.70)	45.59 (0.79)	50.41 (1.01)	54.47 (0.83)	<0.001
Gender (*n*, %)						<0.001
Female	1952 (50.86)	411 (39.52)	472 (51.63)	500 (53.41)	569 (61.20)	
Male	1898 (49.14)	563 (60.48)	491 (48.37)	455 (46.59)	389 (38.80)	
Race (*n*, %)						<0.001
Mexican American	454 (7.93)	148 (10.31)	164 (10.83)	91 (6.45)	51 (3.17)	
Non-Hispanic Black	775 (10.80)	154 (8.90)	195 (11.15)	227 (12.76)	199 (10.64)	
Non-Hispanic White	1712 (68.91)	527 (72.08)	432 (67.37)	380 (66.83)	373 (69.01)	
Other Hispanic	355 (5.55)	72 (4.49)	89 (5.95)	116 (7.17)	78 (4.66)	
Other Race	554 (6.82)	73 (4.23)	83 (4.70)	141 (6.79)	257 (12.51)	
Education (*n*, %)						<0.001
Below high school	801 (15.39)	246 (20.09)	218 (16.61)	181 (13.50)	156 (10.19)	
High school graduate	804 (19.84)	242 (24.02)	201 (18.70)	194 (19.31)	167 (16.58)	
Above high school	2245 (64.78)	486 (55.89)	544 (64.70)	580 (67.19)	635 (73.23)	
PIR (*n*, %)						<0.001
<1	875 (16.29)	308 (23.86)	230 (17.15)	193 (13.67)	144 (8.73)	
≥1	2975 (83.71)	666 (76.14)	733 (82.85)	762 (86.33)	814 (91.27)	
BMI (kg/m^2^)	29.12 (0.19)	28.75 (0.23)	29.74 (0.42)	29.57 (0.36)	28.40 (0.33)	0.04
Smoke (*n*, %)						<0.01
No	2193 (56.80)	480 (49.42)	544 (57.87)	555 (58.83)	614 (62.45)	
Yes	1657 (43.20)	494 (50.58)	419 (42.13)	400 (41.17)	344 (37.55)	
Depression						0.09
No	3516 (92.19)	885 (91.20)	865 (89.96)	884 (93.93)	882 (94.14)	
Yes	334 (7.81)	89 (8.80)	98 (10.04)	71 (6.07)	76 (5.86)	
Alcohol user (*n*, %)						0.89
No	533 (10.59)	118 (10.48)	121 (9.98)	134 (11.16)	160 (10.83)	
Yes	3317 (89.41)	856 (89.52)	842 (90.02)	821 (88.84)	798 (89.17)	

*PIR* poverty income ratio, *BMI* body mass index

### Multivariate logistics regression analyses

Table 2 presents the OR and 95% CI of depression for quartiles of DHA levels, using the lowest quartile category as the reference. In Crude, unadjusted for any factor, depression was correlated with DHA in the highest quartile (OR = 0.644, 95% CI 0.419–0.991). In Model 1, adjusted for age, gender, and race, the third (OR = 0.542, 95% CI 0.316–0.930) and highest quartiles (OR = 0.478, 95% CI 0.287–0.797) for DHA. In Model 2, additional adjustments were made for PIR, BMI, education level, and smoking and drinking status, and although no significant correlations were observed, the trend in risk of depression with DHA levels persisted (*P* for trend = 0.04).

**Table 2:** Weighted multivariable-adjusted logistic regression for risk of depression in NHANES 2011–2014

	Crude OR (95% CI)	*p* value	Model 1 OR (95% CI)	*p* value	Model 2 OR (95% CI)	*p* value
DHA						
DHA level (μmol/l)	0.998 (0.995, 1.000)	0.05	0.996 (0.993, 0.999)	0.01	0.998 (0.995, 1.001)	0.11
Categories						
Q1	Reference		Reference		Reference	
Q2	1.156 (0.682, 1.960)	0.58	1.019 (0.599, 1.734)	0.94	1.159 (0.653, 2.054)	0.60
Q3	0.669 (0.400, 1.121)	0.12	0.542 (0.316, 0.930)	0.03	0.646 (0.393, 1.064)	0.08
Q4	0.644 (0.419, 0.991)	0.05	0.478 (0.287, 0.797)	0.01	0.663 (0.395, 1.112)	0.11
*p* for trend		0.01		<0.01		0.04

Crude: no covariates were adjusted

Model 1: age, gender, and race were adjusted

Model 2: age, gender, race, PIR, BMI, education, alcohol, and smoking were adjusted

*OR* odds ratio, *CI* confidence interval

### Causal effect of DHA on depression

**Fig. 2:**
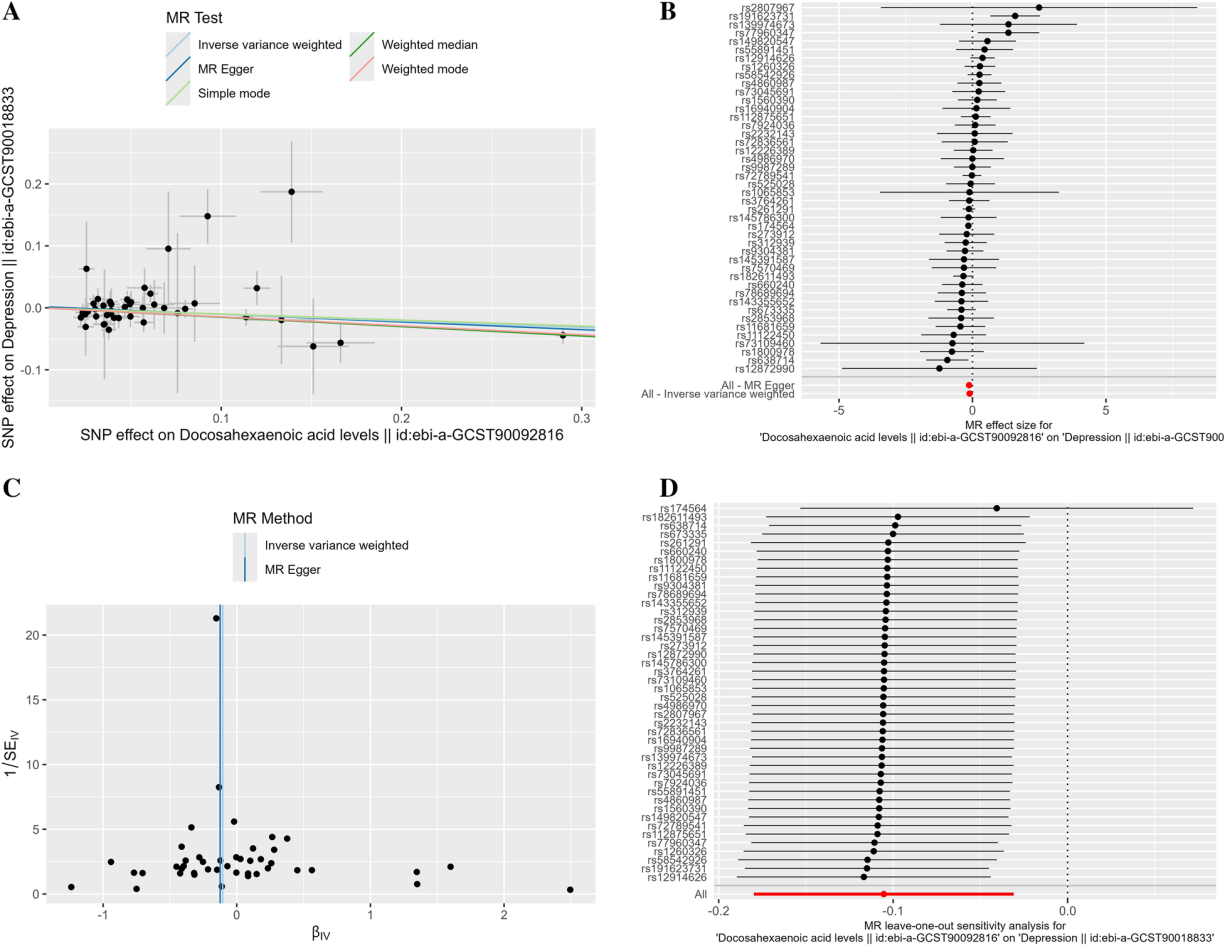
MR estimates of the association between DHA and depression. **A** Scatter plots of MR analyses. **B** Forest plot shows the results of each MR estimate. **C** Funnel plots show no significant heterogeneity among the SNPs. **D** Forest plot of leave-one-out sensitivity analysis shows the impact of each SNP on the overall causal estimate. MR, Mendelian randomization; SNPs, single-nucleotide polymorphisms

We chose 44 SNPs as IVs for the DHA cohort (ebi-a-GCST90092816). All IVs were not significantly associated with depression or any confounding factors. In addition, we calculated the F-statistic for each SNP. The F-statistics of these IVs ranged from 30.282 to 4,996.611, indicating that the IVs were unlikely to be influenced by instrument bias. Finally, MR analysis was performed on the depression cohort (ebi-a-GCST90018833). IVW was used as the primary analysis method. As shown in Table 3, the results indicated a causal relationship between genetically predicted DHA levels and reduced risk of depression (OR = 0.900, 95% CI 0.835–0.970, *P* = 0.006). The scatter plot and forest plot are shown in Fig. 2A, B.

**Table 3:** MR estimates for the association between genetically predicted DHA and depression risk

Analytical methods	OR	95% CI		*p* value
		Lower limit	Upper limit	
MR–Egger	0.883	0.793	0.984	0.030
Weighted median	0.860	0.787	0.939	0.001
Inverse variance weighted	0.900	0.835	0.970	0.006
Simple mode	0.907	0.700	1.176	0.464
Weighted mode	0.865	0.798	0.938	0.001

### Sensitivity analyses and validation of MR

According to the results of Cochran’s Q test, we used IVW and MR–Egger regression to untested heterogeneity (*P* = 0.244, 0.219, respectively). The funnel plot (Fig. 2C) demonstrated symmetry, which further suggested that there was no heterogeneity. The MR–Egger regression method’s intercept *p* value was 0.637 (>0.05), which suggests that there is no horizontal pleiotropy among IVs. The MR–PRESSO method yielded a result of *P* = 0.265, which supported this finding. In addition, leave-one-out was performed to exclude the effect of individual SNPs on the overall causal estimates (Fig. 2D).

Combined with the results of sensitivity analyses, the credibility of causal conclusions was elevated, with low heterogeneity and no pleiotropy, supporting that the original causal estimates of IVW were not disturbed by confounding or bias. The leave-one-out method confirms the stability of the results and excludes the effect of individual SNP anomalies.

### Causal effect of depression on DHA

We also examined the reverse effect of depression on DHA using the IVs associated with depression. The results showed that, excluding the possibility of reverse causality, the causal impact of depression on DHA was not statistically significant (IVW: OR = 1.007, 95% CI 0.990–1.024, *P* = 0.421). The detailed results of the five MR methods are shown in Table S2. Reverse MR analyses ruled out the interference of reverse causality on the main analysis’s results. This result is consistent with biological mechanisms that DHA metabolism is mainly regulated by genetics and long-term dietary patterns, and the behavioral effects of depression make it difficult to significantly alter its levels. Combining the main analysis with the sensitivity analysis, we are more confident that the protective effect of DHA on depression is causal.

## Discussion

Consistency between cross-sectional study results and MR analysis further clarified the association between DHA and depression, showing DHA to be a protective factor against depression.

There are currently many studies supporting the association between DHA levels and depression, but there are different results for different study populations. Emery [31] conducted an RCT on 107 adolescents aged 8–17 with moderate to severe depression. Compared with patients with moderate depression, patients with severe depression had significantly higher levels of DHA and n-3 PUFAs status, and believed that this may be due to changes in the nutritional patterns of severely depressed youth compared to those with moderate depression. In a longitudinal study from the Cooper Center that included adults aged 20–90 years [32], DHA levels were significantly correlated with depression scale scores, suggesting that DHA levels were associated with the severity of depressive symptoms, and that an association with n-3 PUFAs supplement use habits was considered. It was also noted that lifestyle and/or other environmental factors unrelated to DHA levels may also be associated with the severity of depressive symptoms, and the role of health-related mediators in these relationships needs to be assessed. The results of another recent national cross-sectional study also showed [33] that n-3 PUFAs (EPA and DHA) levels predicted the onset and severity of cognitive impairment in elderly patients with depression, but a causal relationship could not be drawn due to the cross-sectional design.

In this study, we investigated the causal association between DHA and depression by combining a MR study with a large, nationally representative observational study, the NHANES. Regardless of whether we consider DHA levels as a continuous variable or a categorical variable, observational analyses indicate that after adjusting for confounding factors, DHA levels can, to some extent, reduce the risk of depression (Table 2). Based on our findings, DHA has a moderate protective effect against depression, which is slightly different from many previous studies. This modest protective effect of DHA may be influenced by the progression of depression severity. According to the 2021 Cochrane Systematic Review [34], based on 35 studies involving 1,964 participants, there is insufficient evidence to support the effect of n-3 PUFAs on major depression in adults. Only small to moderate non-clinically significant effects were detected in alleviating depressive symptoms. Another MR study noted that high concentrations of EPA in brain tissue were more able to alleviate neuroinflammation compared to DHA [35]. Age may also play a role, and a recent meta-analysis by Chang et al. [13] also suggests that n-3 PUFAs can alleviate depressive symptoms in elderly patients with dementia. Most likely, in depression, n-3 PUFAs are not sufficient as monotherapy to treat this disorder, but these nutrients can be used as adjunctive therapy to standard antidepressants [36]. Perhaps for the associations observed in this study, there may be more pronounced effects in specific subgroups (e.g., dietary patterns). In a recently published meta-analysis, data from prospective studies showed that adherence to the Mediterranean diet was associated with fewer self-reported depressive symptoms [37]. A Japanese population-based cohort study suggested that higher n-3 PUFAs intake may be associated with a lower risk of depressive symptoms in populations with high fish consumption [38].

It is important to note, though, that the association between the two was not statistically significant in Model 2, but the trend remained. For this result, similar to the study by Wang et al. [22], they also observed that the association between plasma DHA and depression did not persist after controlling for all potential confounding factors. This may be due to the source of the DHA samples. The DHA data we chose were all DHA concentrations in plasma samples obtained at a single time point, and plasma FAs containing DHA may not fully capture the components of FAs present in erythrocytes or phospholipid fractions. Erythrocyte membrane FAs have been reported to reflect long-term effect states [39], but the NHANES database does not have this type of data, so we suspect that the loss of significance may be only temporary; after all, the trend toward correlation remains. In addition, the presence of residual confounding factors is possible. In addition, in Wang et al.’s study [22], reference was made to the competing effects of arachidonic acid (AA) with DHA, so they turned to utilizing AA/DHA ratios to study associations with depression in the context of observing the disappearance of significance of a single DHA level in association with depression. In addition, in the process of selecting research subjects, we deleted some research populations with missing data, which may result in an insufficient sample size. In the future, more research populations need to be included for further verification.

Despite adjusting for measured confounders, potential bias may still exist. Therefore, additional validation is required to establish the causal association between DHA and depression. MR can overcome the problem of unknown confounding and reverse causation in traditional observational studies [16]. In this study, we used a cohort of patients from the GWAS database to evaluate DHA and depression risk. A causal association between DHA and a lower incidence of depression is supported by the findings of four additional methodologies, as well as IVW (Table 3). Our observational study, based on Americans and MR on Europeans, demonstrates that DHA may indeed be a protective factor for reduced depression in different populations. However, it cannot be denied that there is a mismatch between the populations in the NHANES (American Adults) and GWAS (European Ethnicity) databases, particularly due to differences in population stratification caused by racial composition, and dietary habits in different regions, especially fish consumption, affecting the differences in DHA intake between the United States and Europe [40]. According to the latest survey on global levels of n-3 PUFAs, the United States is at a lower level, while Europe is mostly at a moderate level [41]. Therefore, it is necessary to pay attention to cross-racial MR and multicenter multi-ethnic cohort studies in the future. Based on our findings, there are implications for other regional populations, suggesting that DHA may play a similar role in reducing the risk of depression. However, the dietary structure and habits of populations in different regions vary greatly. For example, residents of some Asian countries, such as Japan and Korea, have a higher intake of fish, which is an important food source of DHA, and their DHA intake may be different from that of the American population. In some parts of Africa and South America, the intake of DHA-containing foods and supplements may be relatively low due to dietary habits and food resource constraints. Such dietary and cultural differences result in different basal levels of DHA in different populations, which may affect the strength of the association between DHA and depression. Therefore, more large-scale observational studies based on local populations are needed to clarify the true association between DHA and depression in different regional populations. 

In our MR analysis, the range of F-statistics is relatively large (30.282–4,996.611). The F-statistic depends on the SNP’s effect on exposure. If some SNPs have strong effects (such as the main gene) and others have weak effects, the F-statistic will show a larger range. In addition, sample size is a key factor in calculating F-statistic values, especially in two-sample MR, where different exposure and outcome sample sizes may lead to fluctuations in F-statistic values. Although this study used multiple sensitivity analyses to exclude the influence of weak IVs and the pleiotropy of IVs, the differences in sample size and the small number of IVs cannot be avoided.

Although the exact mechanism of DHA and depression is not yet clear, based on recent relevant research, we make the following hypotheses. Brain lesions in depression are mediated by inflammatory processes inside the central nervous system (CNS) [29]. Patients with depression have elevated levels of inflammatory cytokines [30]. DHA has anti-inflammatory properties and regulates various physiological and pathological processes, including inflammation, allergic reactions, blood lipid regulation, and cell metabolism. In Borsini’s study [42], it was first demonstrated that EPA and DHA are metabolized into lipid mediators such as the LOX (5-HEPE and 4-HDHA), CYP450 hydroxylase (18-HEPE and 20-HDHA) and epoxygenase [17(18)-EpETE and 19(20)-EpDPA] in neurons, thereby preventing a decrease in neurogenesis, an increase in neuronal apoptosis, and an increase in inflammatory transcription factors induced by pro-inflammatory cytokines. These identical metabolites are elevated in the plasma of depression patients receiving EPA or DHA treatment, indicating that these lipid mediators are novel molecular mechanisms supporting the antidepressant, anti-inflammatory, and neuroprotective effects of PUFAs. In a recent review[12], Serefko pointed out that COX and LOX enzymes promote the conversion of n-3 PUFAs into various products, including prostaglandins and leukotrienes, as well as monohydroxy and polyhydroxy fatty acids. Meanwhile, cytochrome P450 promotes the conversion of omega-3 polyunsaturated fatty acids into epoxy and hydroxylated lipid derivatives. EPA and DHA both reduce the chemotaxis of neutrophils and monocytes, inhibit the synthesis of pro-inflammatory mediators (i.e., interleukin-1β and TNF-α), and suppress T cell proliferation. In addition, this review suggests that another mechanism may be the role of omega-3 polyunsaturated fatty acids in maintaining membrane stability and flexibility. Lack of DHA levels is associated with neuronal membrane integrity and abnormal signaling of neurotransmitters, including serotonin, dopamine, and norepinephrine. These disorders may lead to the development of emotional and cognitive dysfunction, which is a characteristic of depression. Another potential mechanism is the gut flora. In a review by Costantini [43], it was pointed out that dysbiosis of the Firmicutes/Bacteroidetes ratio is associated with MDD [44], with a decrease in bifidobacteria combined with an increase in Escherichia coli, leading to the establishment of endotoxemia and chronic low-grade inflammation associated with depression [45]. n-3 PUFA can reverse this situation by restoring the Firmicutes/Bacteroidetes ratio and increasing the Firmicutes family. Animal studies have shown that n-3 PUFA can increase the inhibition of lipopolysaccharides (LPS) in bacteria (Bifidobacterium) and reduce the production of LPS in bacteria (*Escherichia coli*), thereby eliminating endotoxemia. Future animal studies on the inhibitory effects of DHA on neuroinflammation and gut microbiota, as well as human RCTs linking changes in DHA, inflammation, and gut microbiota to improvements in depression through multi-omics analyses, are still needed to demonstrate this.

The two strengths of the current study are as follows. On one hand, we integrated MR analysis with observational investigations. Studies that just use observation are vulnerable to unmeasurable confounders. Even after adjusting for confounding variables, MR by itself has a comparatively high false-negative rate. When we combine these two approaches, the consistency of the outcomes increases the validity of our conclusions. On the other hand, our MR analysis had enough statistical power to quantify the relationship between DHA and depression, because it was based on a large amount of data that had undergone rigorous sensitivity analysis and validation.

This study still has certain limitations. First of all, assessment of depression by the PHQ-9 self-rating scale may introduce subjective bias and misclassification, and it is not possible to analyze the severity or subtypes of depression due to limited data. Second, the NHANES database only included people ≥20 years of age; therefore, we were unable to investigate the possibility of depression in children and adolescents. Third, due to restrictions on the genetic data, we were unable to do additional subgroup analyses in the MR analyses. Fourthly, despite the high F-statistics, there may be weak instrumental bias in MR. Although a variety of MR methods have been used in bidirectional studies, residual multidirectionality may still be present. Moreover, there is a possibility of residual confounding in NHANES and GWAS data; socioeconomic status, dietary patterns, and co-morbidities and medications were not fully adjusted for in observational studies; population stratification was a confounding factor at the genetic level in MR analyses. Lastly, our observational study data are from the United States, and the MR study data are from Europe, which not only limits the applicability of the present findings to other population groups, but also the population differences between the two types of studies need to be corroborated by subsequent studies.

## Conclusion

This study combines a large nationally representative observational study in NHANES and MR analysis surveys to provide various evidence for a protective association between DHA levels and depression. Large, well-documented RCTs and MR studies in different populations are also needed to confirm causality.

## Supplementary Information

Additional file 1.

## Data Availability

No datasets were generated or analysed during the current study.
