# Supplementary material for: Association between DHA and depression: results from the NHANES 2011–2014 and a bidirectional Mendelian randomization analysis
Source: Eur J Med Res. 2025 Jul 22;30:656. doi: 10.1186/s40001-025-02918-4 (PMC12281733; doi:10.1186/s40001-025-02918-4)
Supplement: Supplementary file 1 — Additional file 1. [file 40001_2025_2918_MOESM1_ESM.docx]

**Supplementary Materials**

1. Specific details of DHA levels and depression genes

Genetic summary data for DHA were obtained from the Richardson TG study, a GWAS study sample that included a total of 11,5006 European subjects and estimated the genetic association of 11,590,399 SNPs with DHA, which is the largest dataset in terms of sample size for this phenotype. To make the genetic characteristics of the two samples similar, databases with European populations were uniformly selected for the study, thus avoiding bias due to population stratification. Based on the above criteria, the latest and largest sample size GWAS was selected as the outcome sample. Refer to Table S1 for additional details about GWAS.

Table S1 Information on genetic instruments and outcome source.

| Traits | Data source | PMID | Year | Sample size | GWAS ID |
| --- | --- | --- | --- | --- | --- |
| Docosahexaenoic acid levels | Richardson TG | 35213538 | 2022 | 115,006 | ebi-a-GCST90092816 |
| Depression | Sakaue S | 34594039 | 2021 | 449,414 | ebi-a-GCST90018833 |

2. Formula for calculating F statistic

R^2^ = 2  × EAF × (1 − EAF) × (**β**)^2^

*F* statistic= R^2^ × (N − 2) / (1 − R^2^)

NOTE: R^2^(The variance):the proportion of exposed variability explained by individual genetic instrument; EAF: the effect allele frequency; **β**: the estimated effect of SNP; Ν: the sample size of the GWAS.

3. Reverse MR Study of Depression on DNA

Table S2 Reverse MR analysis of depression and DHA levels using GWAS dataset.

| outcome | exposure | method | nsnp | b | se | pval |
| --- | --- | --- | --- | --- | --- | --- |
| Docosahexaenoic acid levels \|\| id:ebi-a-GCST90092816 | \|\| id:ebi-a-GCST90018833 | MR Egger | 22 | 0.012277564171933 | 0.0110412998405168 | 0.279340583126189 |
| Docosahexaenoic acid levels \|\| id:ebi-a-GCST90092816 | \|\| id:ebi-a-GCST90018833 | Weighted median | 22 | 0.00724296025802671 | 0.0106676656629022 | 0.497160713462092 |
| Docosahexaenoic acid levels \|\| id:ebi-a-GCST90092816 | \|\| id:ebi-a-GCST90018833 | Inverse variance weighted | 22 | 0.00679014078389493 | 0.00844282753971389 | 0.421252830955364 |
| Docosahexaenoic acid levels \|\| id:ebi-a-GCST90092816 | \|\| id:ebi-a-GCST90018833 | Simple mode | 22 | 0.010315282550261 | 0.0248883574527148 | 0.682738761421226 |
| Docosahexaenoic acid levels \|\| id:ebi-a-GCST90092816 | \|\| id:ebi-a-GCST90018833 | Weighted mode | 22 | 0.00752017654106985 | 0.0106074733120921 | 0.486151770777344 |
